# Distance of the alveolar antral artery from the alveolar crest. Related factors and surgical considerations in sinus floor elevation

**DOI:** 10.4317/medoral.21475

**Published:** 2016-10-01

**Authors:** Pablo Varela-Centelles, María Loira-Gago, Antonio Gonzalez-Mosquera, Juan M. Seoane-Romero, José M. Garcia-Martin, Juan Seoane

**Affiliations:** 1 C.S. Praza do Ferrol. EOXI Lugo, Cervo e Monforte de Lemos. Galician Health Service. Pza. Ferrol 11. 27001 Lugo. Spain; 2 Stomatology Department. School of Medicine and Dentristry. University of Santiago de Compostela. Entrerríos s/n. 15782. Santiago de Compostela. A Coruña. Spain; 3 Department of Medicine and Medical-Surgical Specialities. School of Medicine and Health Sciences. University of Oviedo. C/ Julián Clavería s/n. 33006. Oviedo. Spain

## Abstract

**Background:**

In a variable proportion of maxillary sinuses alveolar antral artery is located close to the residual ridge, increasing the chances for haemorrhagic complications during sinus floor elevation procedures.

**Material and Methods:**

Retrospective observational study of CBCT explorations performed for implant-treatment planning. The upper first molar area was selected for this study. The relative uncertainty (standard deviation of the measurement divided by its mean and expressed as a percentage from 0% to 100%) was chosen for determining the observational errors. For modeling the chances of AAA detection, the generalized additive models (GAM) approach was chosen.

**Results:**

A total of 240 maxillary sinuses were studied (46.25% males) whose median median age was 58 years old (IQR: 52-66). Univariate models showed that the chances for an AAA-alvelar crest distance ≤15mm increase in wider sinuses with lower, subsinusally edentulous crests. When distance is considered as a continuous variable, the best mutivariate model showed an explained deviance of 67% and included AAA diameter, distance AAA-sinus floor, sinus width, and shape, height and width of the residual ridge. Thinner AAAs are found closer to the crest (within the ≤15mm safe distance).

**Conclusions:**

Bearing in mind the inclusion criteria and the limitations of this investigation, it is concluded that there is a high proportion of maxillary sinuses where AAA describes a course close to the alveolar crest (≤15mm), which was classically considered a safe distance for SFE. This position is related to the presence of atrophic crests (depressed ridge form) and wide maxillary sinuses where the distance of the vessel to the floor of the sinus is small. This information may permit a better surgical planning of SFE procedures.

**Key words:**Cone-beam computed tomography, blood vessels, sinus floor augmentation, intraoperative complications.

## Introduction

Although maxillary sinus floor elevation (SFE) by lateral approach is a safe and predictable surgical technique for gaining bony tissue for implant placement, it is not free from intra- and post-operative complications (1,2). The second most frequent of these complications (right after sinus membrane perforation) is bleeding secondary to surgical damage to the alveolar antral artery (AAA) during antrostomy (2).

AAA is an anastomosis of the posterior superior alveolar artery (PSAA) and the infraorbital artery (IOA) located at the anterolateral wall of the sinus with a variable course, which supplies the Schneiderian mebrane, the sinus wall, and the periosteum (3). It also contributes to graft integration and to the healing of the surgical wound (2).

A careful surgical planning using cone beam computed tomography (CBCT) has been advocated in an attempt to minimise bleeding complications during SFE. Yet, AAA detection rate by CBCT is 78.1% (95%CI=61.2-94.9) (3), and it has proved unable to disclose small arteries (<0.5mm) and those with a intrasinusal or fully extraosseous course (2,3).

In any case, the vascularity of the lateral sinus wall varies widely among individuals (2), being the risk for intrasurgical bleeding higher for larger vessels (>2mm) (2), in severely atrophic ridges, and at the area of the first upper molar where the distance between AAA and the alveolar crest is shorter (4,5).

The upper limit of the antrostomy is directly related to the length of the implant to insert (6,7), and 15mm from the bony crest is considered a safe distance to prevent vascular damage (4,8,9). However, in a variable proportion of maxillary sinuses AAA is located closer to the ridge (9) increasing this way the chances for haemorrhagic complications, particularly for patients whose AAA diameter ranges from 1 to 2 mm where the risk for bleeding could reach 57% (10). Despite this fact, there are no studies focused on identifying factors explaining AAA course variations at the anterolateral sinus wall invading the aforementioned safe-distance. Thus, the aim of this investigation was to identify and model the variables related to patients at risk of iatrogenic bleeding during SFE whose AAAs are located within 15mm of the bony crest.

## Material and Methods

In order to attain the aims of this investigation, a retrospective observational study was designed that met the requirements of the university’s Ethics Committe. After obtaining informed consent, the study was carried out from March to November 2015 at the Radiology Unit of the School of Medicine and Dentistry of the University of Santiago de Compostela (Spain).

CBCT explorations were identified using the unit’s database, and retrieved if fulfilled the inclusion criteria, namely explorations performed for implant-treatment planning in maxillary edentulous or subsinusally edentulous patients, or subjects with (an) upper first molar(s) missing. Exclusion criteria were: poor image quality, sinus disorders or previous history of sinus surgery or grafting. As a result, 240 maxillary sinuses with clearly defined AAA were selected out of 466 sinuses that met the inclusion criteria.

All patients were explored by means of a cone beam CT (I-CAT, 17-19. Imaging Sciences International, 1910 North Penn Toad, Hatfield, USA) with its I-CAT software (Imaging Sciences International) set at a resolution of 0.3 voxels with 8.9 seconds of capture time. According to previous findings (4,5), the course of AAA lies closer to the bony crest at the upper first molar level, so this location was selected for performing the coronal sections used in this study.

Two researchers (ML & AG) used the resources provided by the proprietary software (I-CATvision) to undertake linear measurements in CBCT explorations.

The relative uncertainty (standard deviation of the measurement divided by its mean and expressed as a percentage from 0% to 100%) was chosen for determining the observational errors. The variables considered (with its relative uncertainty) included: distance from AAA to sinus floor (0%), and distance from AAA to alveolar crest (0%).

The outcome variable was “distance of AAA from the bony crest”, considered both as continuous and dichotomous (coded as “1” for distances ≤15mm -risk for iatrogenic damage- and “0” for higher values). Additional potentially-related co-variates, such as gender, pattern of edentulism (maxillary edentulous, subsinusally edentulous, or upper first molar missing), and course of the artery (fully intraosseous; intrasinusal -between the Schneiderian membrane and the sinus bony wall-; or superficial -on the outer cortex of the lateral sinus wall) were also studied. The residual ridge was categorised according to Cawood and Howell (11) as class II: immediately post extraction; class III: well-rounded ridge; class IV: knife-edge ridge; class V: flat ridge; and class VI: depressed ridge form. Other variables considered in this investigation were: thickness of the lateral sinus wall, height of the residual bony crest, width of the residual alveolar ridge (at the basal and crestal levels) and width of the maxillary sinus.

- Statistical analysis

The study unit for this research was not the patient, but the maxillary sinus. Descriptive statistics are summarised using frequencies for categorical variables and the median and mean (central trend statistics), together with the inter-quartile range (IQR) as spread indicant for the quantitative ones.

For modeling the chances of AAA detection, the generalized additive models (GAM) approach was chosen, as it permits modeling the effect of co-variates in a flexible manner. The best multivariate model is automatically selected.

All analyses were undertaken using the R software (R Core Team, 2015) with the mgv package for GAM modeling.

## Results

A total of 240 maxillary sinuses were studied (111 males, 46.25%, and 129 females, 53.75%). The median age of the participants was 58 years old (IQR: 52-66). The median width of the sinus lateral bony wall was 11.5 mm (IQR: 9.5-4.1), and AAA mostly described a fully intraosseous course within it (n=117; 48.7%) with diameters >1mm in most cases (80.5%).

The main subsinusal bone resorption patterns were the “well-rounded”(class III: 106; 44.17%) and “flat ridge” (class V: 52; 21.63%) types, with less frequent presentations of class II: 21 (8.75%); class IV: 30 (12.5%); and class VI: 31 (12.9%). The median ridge height was 7.40 mm (IQR: 5.27-9.85) ( Table 1).

**Table 1:**
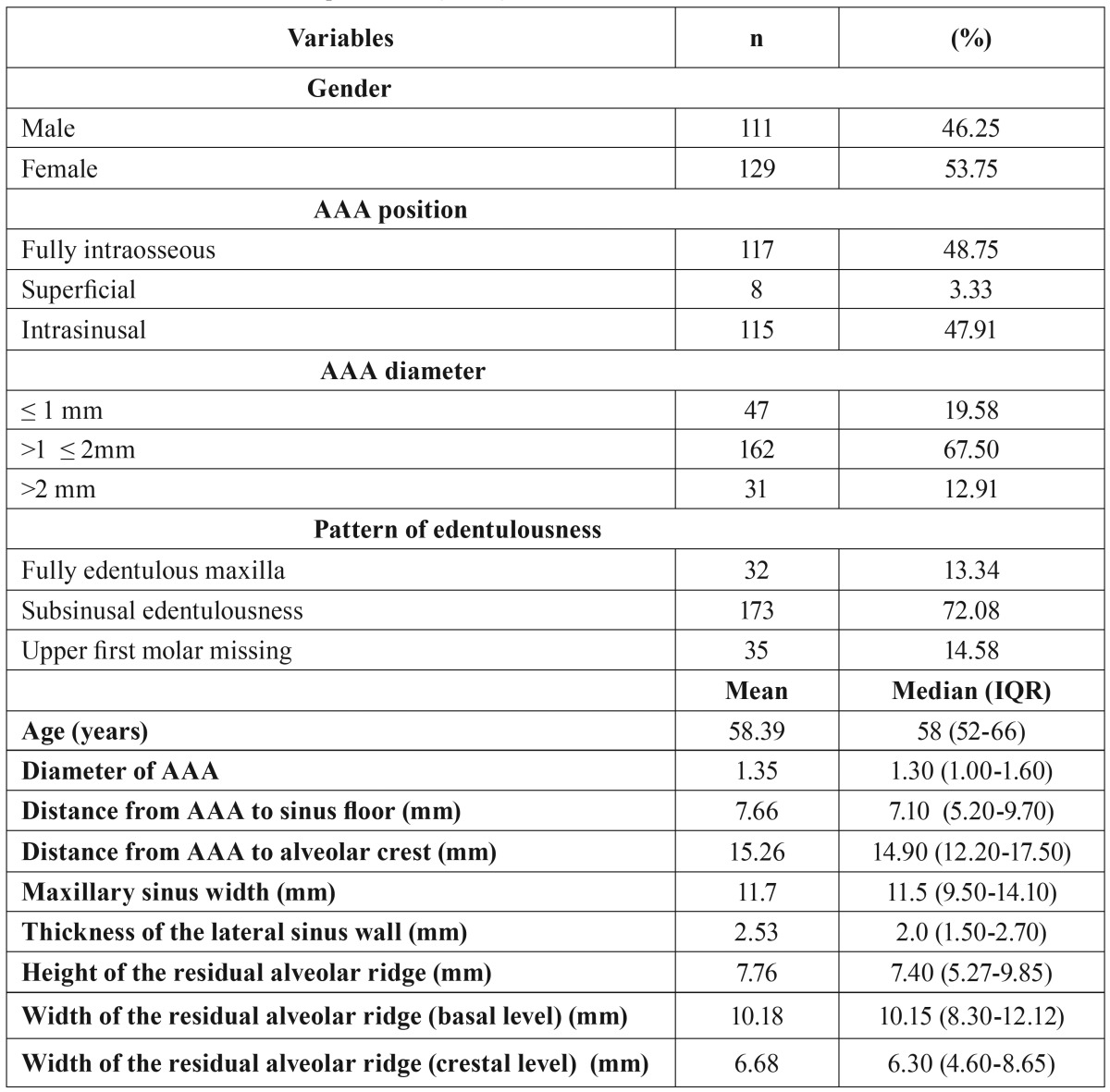
Main features of the sample studied (n=240).

Univariate models have shown a relationship between the AAA-alveolar crest distance and the pattern of edentulousness, bone resorption, height of the residual ridge, distance of AAA to the sinus floor, the sinusal area under the artery, and the width of the maxillary sinus. The chances for an AAA-alvelar crest distance ≤15mm increase in wider sinuses with lower, subsinusally edentulous crests ( Table 2). Yet, when distance is considered as a continuous variable, the best mutivariate model showed an explained deviance of 67% and included AAA diameter, distance AAA-sinus floor, sinus width, and shape, height and width of the residual ridge. (Y= α + β1 ridge shape + f1 (distance from AAA to sinus floor) + f2 (diameter of AAA) + f3 (width of the residual alveolar ridge -basal level-) + f4 (width of the residual alveolar ridge -cresta level-) + f5 (maxillary sinus width), where f1 to f5 represent smooth functions of covariates ( Table 3).

**Table 2:**
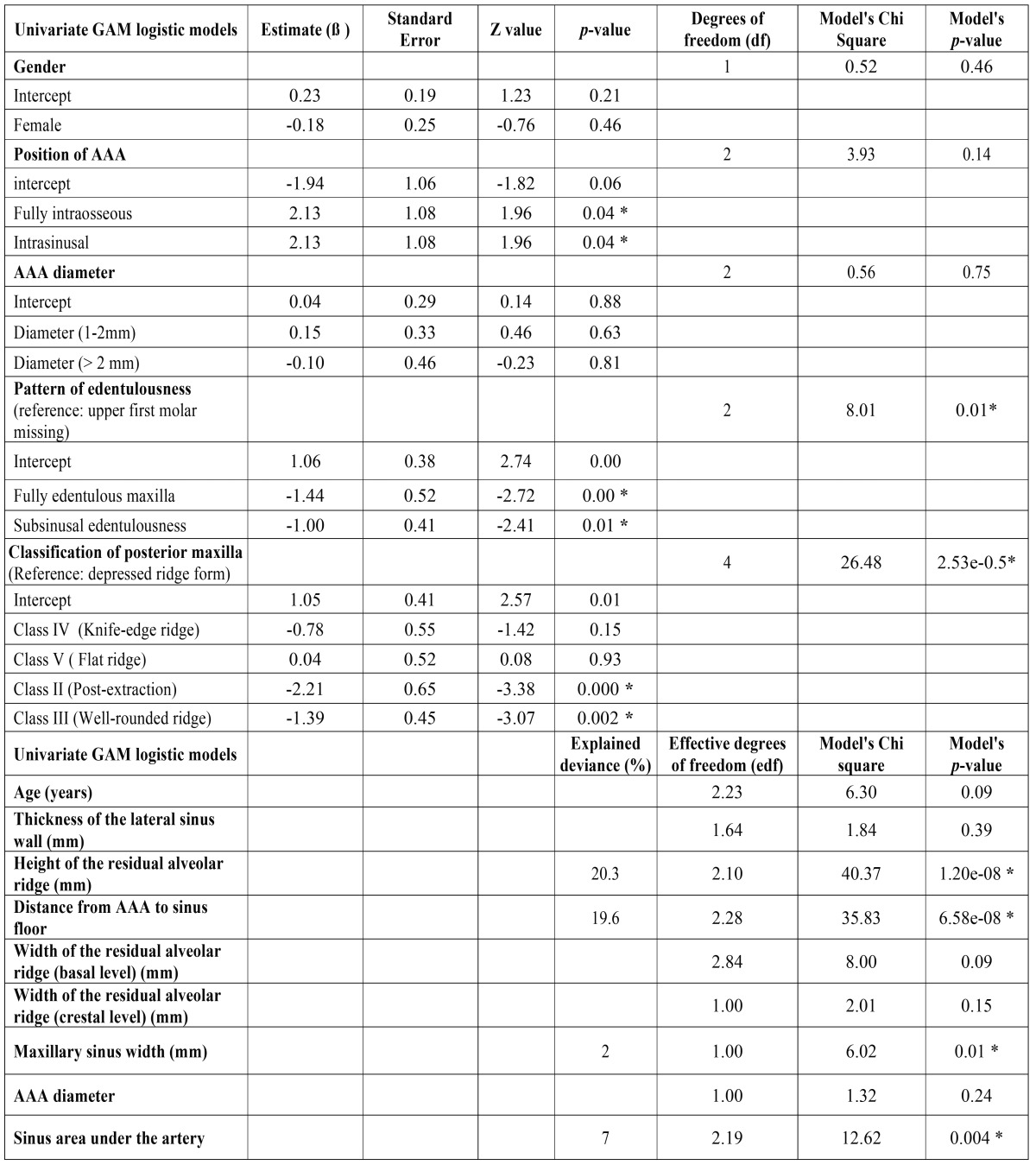
Univariate GAM logistic models for AAA distance to the bony crest (≤ 15mm vs >15 mm).

**Table 3:**
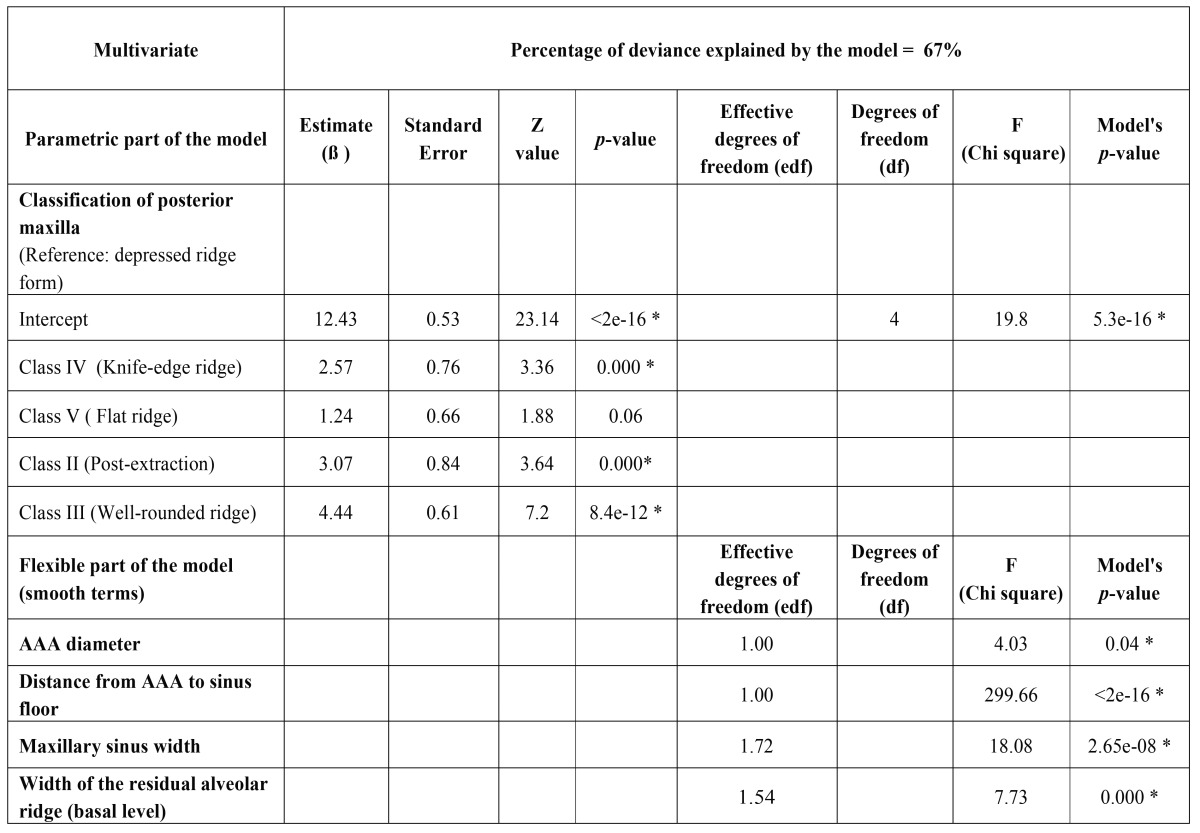
Multivariate model.

**Figure 1:**
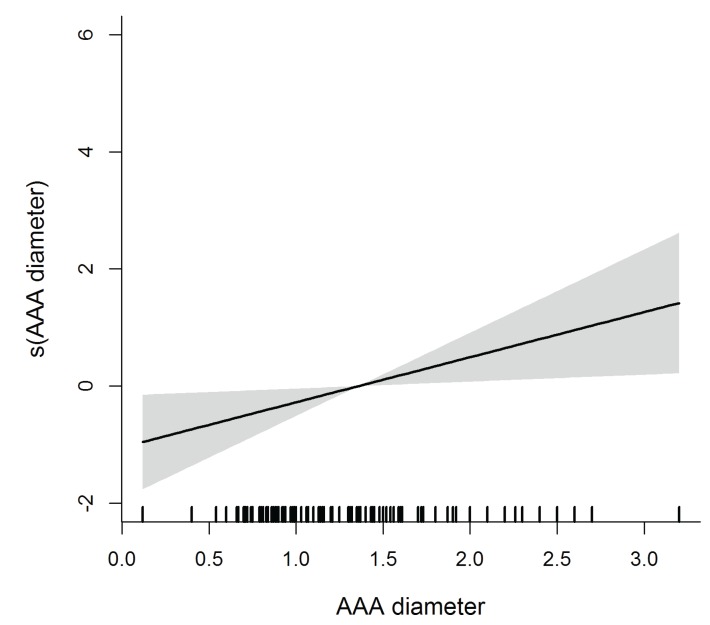
Flexible effect of AAA diameter on the distance of the artery to the alveolar crest.

**Figure 2:**
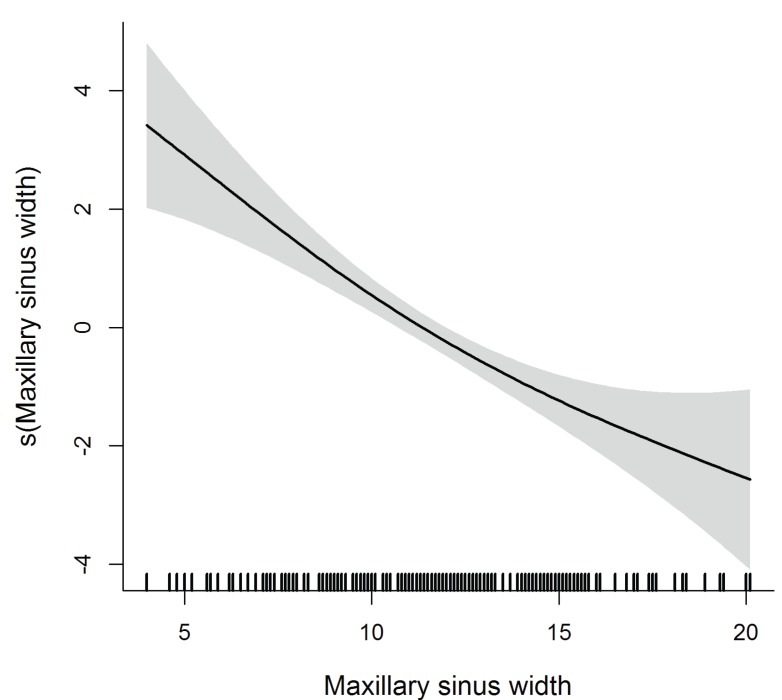
Flexible effect of maxillary sinus width on AAA distance to the alveolar crest.

**Figure 3:**
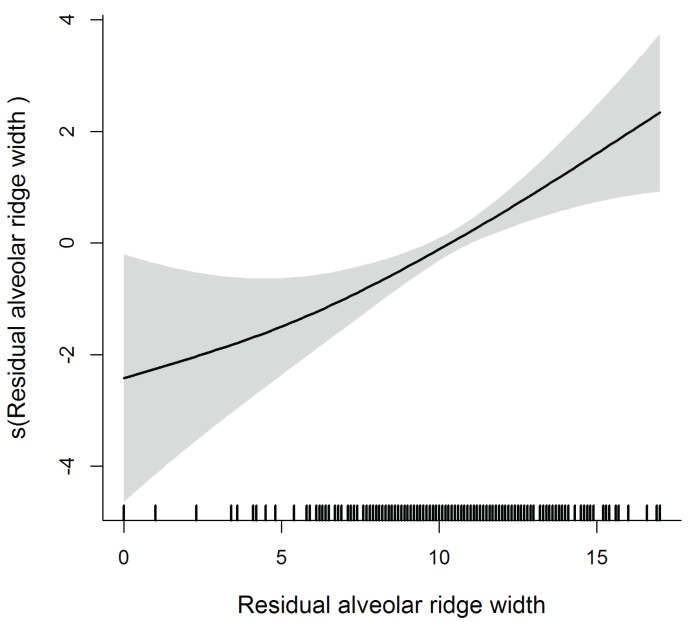
Flexible effect of width of the residual alveolar ridge on AAA distance to the alveolar crest.

The diameter of AAA is significant in the multivariate model, as lager AAAs are found further from the crest whereas thinner AAAs are closer to the crest (within the ≤15mm safe distance). AAA are also closer to the ridge when the distance AAA-floor of the sinus is short and the sinus is wide. Contrarily, ridges with less resorption and thus wider at the basal and crestal levels, are significantly linked to a higher course of the vessel (Fig. 1-3).

## Discussion

Massive bleeding may occur due to accidental vascular damage during SFE by lateral (12,13) or even transcrestal approaches (14). Bleeding then results in longer operative times and favour additional complications such as perforation of the sinus membrane, or reduction of blood supply and displacement of the graft (2). Certain anatomic circumstances, like the diameter and position of the AAA together with the proximity to the alveolar crest may condition the appearance of this kind of complications (3,5). Arteries whose diameter is below 1mm are not a problem (15), but those larger than 2mm may represent a risk for bleeding (2). In this sense, AAA is reported to present an average diameter ranging from 0.9 mm to 15 mm (16). Our findings are consistent with previous reports, with values about 1.3 mm that resulted to be >1 mm in 80% of the sinuses.

The position of the vessel regarding the lateral wall of the sinus also seems to influence the surgical moment with higher risk for haemorhage. Thus, intrasinusal AAAs located between the sinus membrane and the bony wall makes critical the process of membrane detachment (9). This situation has been reported as the second most common (3,5) and accounts for 47.9% in our series. When the vessel follows a superficial course, external to the lateral wall of the sinus, the risk for haemorrhage would be higher when making the incisions, but not only during antrostomy (4). This location does not reach 8% in any case series (9,16-18). Previous reports acknowledge AAA mostly describes a fully intraosseous course in the sinus lateral bony wall (9,16,17), which occurred in 48.7% of the sinuses in our study.

The location of AAA in relation to the alveolar ridge also influences osteotomy, with reported average distances from 11.2 mm (19) to 18.3 mm (20). The variations in extreme values are wider -2.8 mm to 31.7 mm- (9,17), probably due to variations in the height of the residual ridge and in the distance AAA-floor of the maxillary sinus. Our results show a mean value or 15.2 mm for an average AAA-sinus floor distance of 7.6 mm. Besides, and despite a moderate (20-31%) proportion of cases where AAA invades the safe distance of ≤15mm (9,21), our data revealed this phenomenon occurs in up to 48.6% of cases. Thus, this variable should always be taken into account when undertaking SFE procedures.

This investigation has permitted the identification of risk profiles where patients with depressed ridges and sagittally wider sinuses have higher chances for a course of AAA invading the safety distance reported in the literature. On the other hand, patients with rounded and wide alveolar crests whose AAA are identified at more than 6 mm from the sinus floor would be at lower risk for bleeding complications. Moreover, larger arteries tend to locate farther from the bony crest with less probability for iatrogenic damage.

- Surgical recommendations

Surgical planning based upon clinical records and CBCT explorations, and the use of piezoelectric devices for osteotomy permits a reduction of the surgical risk (5). Some authors have suggested ligating arteries >3 mm to prevent severe iatrogenesis (18,20,21). Alternative specific techniques (double window) may also be used to avoid AAA during antrostomy and reducing the risk for bleeding (3,5). In those situations where AAA describes a mainly intrasinusal course, the process of detachment and elevation of the Schneiderian membrane should be particularly careful. When the course of the vessel is fully extraosseous, the risk for bleeding accidents is related to the incision and flap design.

## Conclusions

Bearing in mind the inclusion criteria and the limitations of this investigation, it is concluded that there is a high proportion of maxillary sinuses where AAA describes a course close to the alveolar crest (≤15mm), which was classically considered a safe distance for SFE. This position is related to the presence of atrophic crests (depressed ridge form) and wide maxillary sinuses where the distance of the vessel to the floor of the sinus is small. This information may permit a better surgical planning of SFE procedures.
